# Supplementary material for: Predicting vasovagal reactions to needles from video data using 2D-CNN with GRU and LSTM
Source: PLoS One. 2025 Jan 24;20(1):e0314038. doi: 10.1371/journal.pone.0314038 (PMC11760633; doi:10.1371/journal.pone.0314038)
Supplement: S1 File — S1 Table. An average estimate of model performance across 5-fold splits using pre-trained Xception and ResNet152 with GRU and LSTM on various video lengths in classifying low and high (minority class) VVR groups. S2 Table. The 2D-CNN best model performance on the test split classifying low vs high VVR classes using pre-trained Xception and ResNet152 models with GRU and LSTM on various video sequences ranging from 150 to 25 frames. S3 Table. The obtained F1 score across all tested folds using the shortest video duration (N = 25 frames). (DOCX) [file pone.0314038.s001.docx]

**Supplementary material**

S1 Appendix. To ensure model robustness and reduce the risk of biased performance, we also completed an additional analysis where we used a nested 5-fold stratified validation technique, in which the dataset is divided into 5 subsets, and the model is trained and tested 5 times. In each iteration, one subset is used for testing, and the remaining 4 subsets are used for training. Stratified cross-validation was selected to ensure that each fold contains a representative sample of all classes, which is particularly important due to dataset imbalance. In addition, the data was split based on subject identification number to ensure that the same participants would not appear in both training and testing sets. A nested cross-validation was selected to optimize the hyperparameters and avoid overfitting. We used k=5 for the outer loop (model evaluation) and a k=3 for the inner loop (hyperparameter testing). We evaluated 4 different video lengths ranging from 150 to 25 frames (we excluded 225 frames due to similar performance across tested lengths because our objective is to use the shortest duration possible). In S1 Table we report the average F1 score with standard deviation obtained on the tested split for all tested folds, and in S2 Table we report the results obtained on the best performing model along with used hyperparameters.

S1 Table. An average estimate of model performance across 5-fold splits using pre-trained Xception and ResNet152 with GRU and LSTM on various video lengths in classifying low and high (minority class) VVR groups.

| Model | Number of frames | Average F1 Score across Tested Splits | Standard Deviation of F1 Scores across Tested Splits |
| --- | --- | --- | --- |
| Pre-trained Xception model with GRU | N = 150 | 0.66 | 0.02 |
|  | N = 100 | 0.66 | 0.04 |
|  | N = 50 | 0.51 | 0.25 |
|  | N = 25 | 0.63 | 0.02 |
| Pre-trained Xception model with LSTM | N = 150 | 0.66 | 0.02 |
|  | N = 100 | 0.65 | 0.01 |
|  | N = 50 | 0.66 | 0.02 |
|  | N = 25 | 0.62 | 0.04 |
| Pre-trained ResNet152 model with GRU | N = 150 | 0.68 | 0.01 |
|  | N = 100 | 0.68 | 0.02 |
|  | N = 50 | 0.66 | 0.03 |
|  | N = 25 | 0.67 | 0.03 |
| Pre-trained ResNet152 model with LSTM | N = 150 | 0.68 | 0.02 |
|  | N = 100 | 0.68 | 0.02 |
|  | N = 50 | 0.67 | 0.02 |
|  | N = 25 | 0.67 | 0.02 |

S2 Table. The 2D-CNN best model performance on the test split classifying low vs high VVR classes using pre-trained Xception and ResNet152 models with GRU and LSTM on various video sequences ranging from 150 to 25 frames.

| Model | Number of frames | Precision | Recall | F1 | AUC-PR | MCC | Hyperparameters used |
| --- | --- | --- | --- | --- | --- | --- | --- |
| Pre-trained Xception model with GRU | N = 150 | 0.75 | 0.63 | 0.68 | 0.65 | 0.43 | 'batch_size': 64, 'dropout_rate': 0.1, 'epochs': 100, 'learning_rate': 0.001 |
|  | N = 100 | 0.74 | 0.66 | 0.70 | 0.65 | 0.46 | 'batch_size': 64, 'dropout_rate': 0.1, 'epochs': 100, 'learning_rate': 0.001 |
|  | N = 50 | 0.63 | 0.67 | 0.65 | 0.57 | 0.36 | 'batch_size': 32, 'dropout_rate': 0.1, 'epochs': 200, 'learning_rate': 0.0001 |
|  | N = 25 | 0.71 | 0.61 | 0.66 | 0.61 | 0.40 | 'batch_size': 32, 'dropout_rate': 0.5, 'epochs': 200, 'learning_rate': 0.0001 |
| Pre-trained Xception model with LSTM | N = 150 | 0.76 | 0.63 | 0.68 | 0.65 | 0.45 | 'batch_size': 64, 'dropout_rate': 0.5, 'epochs': 100, 'learning_rate': 0.0001 |
|  | N = 100 | 0.63 | 0.71 | 0.67 | 0.57 | 0.38 | 'batch_size': 64, 'dropout_rate': 0.1, 'epochs': 100, 'learning_rate': 0.0001 |
|  | N = 50 | 0.68 | 0.71 | 0.69 | 0.60 | 0.43 | 'batch_size': 64, 'dropout_rate': 0.1, 'epochs': 200, 'learning_rate': 0.0001 |
|  | N = 25 | 0.73 | 0.61 | 0.67 | 0.64 | 0.40 | 'batch_size': 32, 'dropout_rate': 0.1, 'epochs': 100, 'learning_rate': 0.0001 |
| Pre-trained ResNet152 model with GRU | N = 150 | 0.69 | 0.68 | 0.69 | 0.61 | 0.44 | 'batch_size': 64, 'dropout_rate': 0.5, 'epochs': 200, 'learning_rate': 0.001 |
|  | N = 100 | 0.75 | 0.68 | 0.71 | 0.66 | 0.48 | 'batch_size': 64, 'dropout_rate': 0.1, 'epochs': 100, 'learning_rate': 0.001 |
|  | N = 50 | 0.78 | 0.64 | 0.70 | 0.67 | 0.49 | 'batch_size': 32, 'dropout_rate': 0.5, 'epochs': 100, 'learning_rate': 0.001 |
|  | N = 25 | 0.65 | 0.79 | 0.71 | 0.61 | 0.41 | 'batch_size': 32, 'dropout_rate': 0.5, 'epochs': 200, 'learning_rate': 0.001 |
| Pre-trained ResNet152 model with LSTM | N = 150 | 0.73 | 0.81 | 0.71 | 0.60 | 0.40 | 'batch_size': 64, 'dropout_rate': 0.5, 'epochs': 100, 'learning_rate': 0.001 |
|  | N = 100 | 0.67 | 0.71 | 0.69 | 0.61 | 0.38 | 'batch_size': 64, 'dropout_rate': 0.5, 'epochs': 100, 'learning_rate': 0.001 |
|  | N = 50 | 0.75 | 0.63 | 0.69 | 0.66 | 0.44 | 'batch_size': 64, 'dropout_rate': 0.5, 'epochs': 200, 'learning_rate': 0.001 |
|  | N = 25 | 0.77 | 0.63 | 0.70 | 0.67 | 0.47 | 'batch_size': 64, 'dropout_rate': 0.1, 'epochs': 100, 'learning_rate': 0.001 |

S2 Appendix. We conducted the Friedman test to compare the performance of four machine learning models (ResNet152 with GRU, ResNet152 with LSTM, Xception with GRU, Xception with LSTM) across all tested folds (see S3 Table for all F1 scores across tested folds). The Friedman test statistic was found to be 12.12 with p value of 0.007, indicating significant differences across tested folds. Nemenyi post hoc test revealed that ResNet152 with GRU performed significantly better than Xception with GRU (p = 0.04). No other statistically significant differences were found between other tested models.

S3 Table. The obtained F1 score across all tested folds using the shortest video duration (N = 25 frames).

| 25 frames / 5s | F1 score | | | | |
| --- | --- | --- | --- | --- | --- |
|  | Fold 1 | Fold 2 | Fold 3 | Fold 4 | Fold 5 |
| ResNet152 with GRU | 0.66 | 0.69 | 0.68 | 0.71 | 0.67 |
| ResNet152 with LSTM | 0.71 | 0.66 | 0.65 | 0.70 | 0.67 |
| Xception with GRU | 0.63 | 0.64 | 0.60 | 0.66 | 0.65 |
| Xception with LSTM | 0.63 | 0.60 | 0.65 | 0.57 | 0.67 |
