# Supplementary material for: Predicting vasovagal reactions to needles from video data using 2D-CNN with GRU and LSTM
Source: PLoS One. 2025 Jan 24;20(1):e0314038. doi: 10.1371/journal.pone.0314038 (PMC11760633; doi:10.1371/journal.pone.0314038)
Supplement: Attachment — Submitted filename: Response to Reviewers.docx [file pone.0314038.s002.docx]

Dear editor and reviewers,

We thank both reviewers and the editor for their careful consideration of the manuscript, and for their knowledgeable and constructive comments. We’ve addressed all your suggestions for improvements. You can find a summary of our improvements in relationship to each comment below (marked in red).

Editor comments.

**1. The paper is rather organised in a highly complicated way. It is very difficult to know what are various subsections are under each section. This makes reading a bit difficult for a reader. For a journal article, this needs to be done in a manner that reads like a good scientific story. The introduction seems all over the play, although connected to some extent. It can be improved with different subsections in place.**

We have adjusted the names of the subsections and adjusted the introduction to improve the readability.

**2. I am not quite sure whether ethics approval should come directly within the methods section or should be moved to the section right after the conclusion. Please follow the journal guidelines while preparing the manuscript.**

Thank you for raising this question. We verified that the ethics approval should be included in the methods section as the journal guidelines state that “methods sections of papers on research using human subjects or samples must include ethics statements” (<https://journals.plos.org/plosone/s/human-subjects-research>). However, in the revised manuscript we include the ethics statement after the description of the participants.

**3. It is also better to provide a dataset summary table that can reduce a lot of text or the texts can be moved to the table's caption. I would like to see a panel figure that visually describes each stage of the data collection methodology or the entire pipeline.**

We have provided the entire pipeline from data collection stage to data analysis (see Figure 1).

Fig. 1. An overview of the data collection and used methodology for data analysis. Stage 1: Data collection shows an overview of the blood donation testing procedure and stages. At each of the seven stages, donors reported their VVR levels, and a video recording was made. At each stage, the video recording lasted 1 to 2 minutes. From stage 4 until stage 6, the recording was continuous, lasting between 5 and 27 minutes. *The procedure is slightly different per BCC. At two locations, donors were brought directly to the donation chair after the physician check and hence no recording at stage 3 was made. If donors had to return to the waiting area after the physician check, they would be asked for an additional self-reported VVR score. An additional recording of 1 to 2 minutes would be made. Stage 2: Data preprocessing shows an overview of the video preprocessing steps. At each step, the earliest frames were removed. i.e. the frames closer to self-report VVR measures are kept for the analysis. In addition, the number of frames was reduced from 30 FPS to 5 FPS. Stage 3: Data analysis shows an overview of model training and evaluation steps.

**4. For datasets of this kind, it is very important that the samples are distributed very rationally into train/validation/test sets. Sometimes it becomes a matter of cherry-picking to show a better performance. To have a better estimate of the performance, it is preferred, in such small datasets, to perform a k-fold cross-validation (k=5 is usually good) and provide an average estimate of the performance. This doesn't overestimate nor underestimate the model. Furthermore, since this is a highly skewed dataset, such an experiment is warranted. The authors might want to do that. Please also ensure that there is no data leakage from the training to the testing set while doing the modelling. Since this is the first study in this area, it is better to show a good modelling strategy that the research community can follow.**

We agree with your raised concerns. We have followed your advice and re-trained our models using stratified 5-k fold validation technique to ensure that the model performance is not overly optimistic.

“We used a 5-fold stratified validation technique on the training set, in which the dataset is divided into 5 subsets, and the model is trained and tested 5 times. In each iteration, one subset is used for testing, and the remaining 4 subsets are used for training. Stratified cross-validation was selected to ensure that each fold contains a representive sample of all classes, which is particularly important due to dataset imbalance to reduce the risk of biased performance.

We have also separated our data “based on subject identification number to ensure that the same participants would not appear in both training and testing sets.”

In conclusion, our results were consistent across all tested folds and the best performance was in line with our previously reported findings. We hope that this addresses your concerns.

We included the results in the Supplementary Material. We reported the average F1 score with SD as well as the highest precision, recall, F1, PR-AUC, and MCC scores obtained. Finally, we reported the actual scores of F1 across all tested folds on the shortest video duration for all four models and compared the model performance using the non-parametric Friedman test.

S1 Appendix. To ensure model robustness and reduce the risk of biased performance, we also completed an additional analysis where we used a nested 5-fold stratified validation technique, in which the dataset is divided into 5 subsets, and the model is trained and tested 5 times. In each iteration, one subset is used for testing, and the remaining 4 subsets are used for training. Stratified cross-validation was selected to ensure that each fold contains a representative sample of all classes, which is particularly important due to dataset imbalance. In addition, the data was split based on subject identification number to ensure that the same participants would not appear in both training and testing sets. A nested cross-validation was selected to optimize the hyperparameters and avoid overfitting. We used k=5 for the outer loop (model evaluation) and a k=3 for the inner loop (hyperparameter testing). In S1 Table we report the average F1 score with standard deviation obtained on the tested split for all tested folds, and in S2 Table we report the results obtained on the best performing model along with used hyperparameters.

S1 Table. An average estimate of model performance across 5-fold splits using pre-trained Xception and ResNet152 with GRU and LSTM on various video lengths in classifying low and high (minority class) VVR groups.

| Model | Number of frames | Average F1 Score across Tested Splits | Standard Deviation of F1 Scores across Tested Splits |
| --- | --- | --- | --- |
| Pre-trained Xception model with GRU | N = 150 | 0.66 | 0.02 |
|  | N = 100 | 0.66 | 0.04 |
|  | N = 50 | 0.51 | 0.25 |
|  | N = 25 | 0.63 | 0.02 |
| Pre-trained Xception model with LSTM | N = 150 | 0.66 | 0.02 |
|  | N = 100 | 0.65 | 0.01 |
|  | N = 50 | 0.66 | 0.02 |
|  | N = 25 | 0.62 | 0.04 |
| Pre-trained ResNet152 model with GRU | N = 150 | 0.68 | 0.01 |
|  | N = 100 | 0.68 | 0.02 |
|  | N = 50 | 0.66 | 0.03 |
|  | N = 25 | 0.67 | 0.03 |
| Pre-trained ResNet152 model with LSTM | N = 150 | 0.68 | 0.02 |
|  | N = 100 | 0.68 | 0.02 |
|  | N = 50 | 0.67 | 0.02 |
|  | N = 25 | 0.67 | 0.02 |

S2 Table. The 2D-CNN best model performance on the test split classifying low vs high VVR classes using pre-trained Xception and ResNet152 models with GRU and LSTM on various video sequences ranging from 150 to 25 frames.

| Model | Number of frames | Precision | Recall | F1 | AUC-PR | MCC | Hyperparameters used |
| --- | --- | --- | --- | --- | --- | --- | --- |
| Pre-trained Xception model with GRU | N = 150 | 0.75 | 0.63 | 0.68 | 0.65 | 0.43 | 'batch_size': 64, 'dropout_rate': 0.1, 'epochs': 100, 'learning_rate': 0.001 |
|  | N = 100 | 0.74 | 0.66 | 0.70 | 0.65 | 0.46 | 'batch_size': 64, 'dropout_rate': 0.1, 'epochs': 100, 'learning_rate': 0.001 |
|  | N = 50 | 0.63 | 0.67 | 0.65 | 0.57 | 0.36 | 'batch_size': 32, 'dropout_rate': 0.1, 'epochs': 200, 'learning_rate': 0.0001 |
|  | N = 25 | 0.71 | 0.61 | 0.66 | 0.61 | 0.40 | 'batch_size': 32, 'dropout_rate': 0.5, 'epochs': 200, 'learning_rate': 0.0001 |
| Pre-trained Xception model with LSTM | N = 150 | 0.76 | 0.63 | 0.68 | 0.65 | 0.45 | 'batch_size': 64, 'dropout_rate': 0.5, 'epochs': 100, 'learning_rate': 0.0001 |
|  | N = 100 | 0.63 | 0.71 | 0.67 | 0.57 | 0.38 | 'batch_size': 64, 'dropout_rate': 0.1, 'epochs': 100, 'learning_rate': 0.0001 |
|  | N = 50 | 0.68 | 0.71 | 0.69 | 0.60 | 0.43 | 'batch_size': 64, 'dropout_rate': 0.1, 'epochs': 200, 'learning_rate': 0.0001 |
|  | N = 25 | 0.73 | 0.61 | 0.67 | 0.64 | 0.40 | 'batch_size': 32, 'dropout_rate': 0.1, 'epochs': 100, 'learning_rate': 0.0001 |
| Pre-trained ResNet152 model with GRU | N = 150 | 0.69 | 0.68 | 0.69 | 0.61 | 0.44 | 'batch_size': 64, 'dropout_rate': 0.5, 'epochs': 200, 'learning_rate': 0.001 |
|  | N = 100 | 0.75 | 0.68 | 0.71 | 0.66 | 0.48 | 'batch_size': 64, 'dropout_rate': 0.1, 'epochs': 100, 'learning_rate': 0.001 |
|  | N = 50 | 0.78 | 0.64 | 0.70 | 0.67 | 0.49 | 'batch_size': 32, 'dropout_rate': 0.5, 'epochs': 100, 'learning_rate': 0.001 |
|  | N = 25 | 0.65 | 0.79 | 0.71 | 0.61 | 0.41 | 'batch_size': 32, 'dropout_rate': 0.5, 'epochs': 200, 'learning_rate': 0.001 |
| Pre-trained ResNet152 model with LSTM | N = 150 | 0.73 | 0.81 | 0.71 | 0.60 | 0.40 | 'batch_size': 64, 'dropout_rate': 0.5, 'epochs': 100, 'learning_rate': 0.001 |
|  | N = 100 | 0.67 | 0.71 | 0.69 | 0.61 | 0.38 | 'batch_size': 64, 'dropout_rate': 0.5, 'epochs': 100, 'learning_rate': 0.001 |
|  | N = 50 | 0.75 | 0.63 | 0.69 | 0.66 | 0.44 | 'batch_size': 64, 'dropout_rate': 0.5, 'epochs': 200, 'learning_rate': 0.001 |
|  | N = 25 | 0.77 | 0.63 | 0.70 | 0.67 | 0.47 | 'batch_size': 64, 'dropout_rate': 0.1, 'epochs': 100, 'learning_rate': 0.001 |

**5. As a paper focusing on medical domain applications, one would like to see substantial discussion on those and how previous AI or non-AI-based studies have done those.**

In our introduction, we included a section on current interventions used for preventing vasovagal reactions and what are the limitations. For example:

“Applied muscle tension consists of repeated contractions of muscles in the legs and/or abdomen in order to increase blood pressure [11, 13]. Even though research shows that these techniques could work for a subset of donors [13], a meta-analysis suggested that these techniques are insufficient for the majority of donors [11] and do not reduce the rate of syncopic reactions [13].

Other preventive strategies provided by healthcare professionals range from providing extra information, social support or distractions or even administering calming medication such as low doses of benzodiazepines that allow the patients to reduce their anxiety levels (Gebhardt et al., 2018). Although benzodiazepines reduce the number of vasovagal reactions by addressing underlying fear and anxiety, some side effects might make them less favorable as a prevention strategy, especially, as blood draws or immunizations are quick procedures. All previously applied preventive techniques might be effective, however, they are costly in terms of extra time required by the staff. Hence, there is an enormous need for new prevention methods.”

In addition, we included some examples of other papers that used facial video information to estimate the likelihood of experiencing pain, depression, anxiety and other psychological and physiological measures.

“Video recordings of the face contain many types of useful information. For example, it contains information about facial expressions which, even when they are very subtle, enable the detection of anxiety, stress, fear, pain, and vasovagal reactions [33-38]. In addition, the face contains information of head movements, eye-gaze direction, changes in facial colors such as paleness, etc. With the recent developments in the field of deep learning and in particular automatic face analysis [39-41], it has been shown possible to predict not only mental health conditions such as depression, anxiety, or obsessive-compulsive disorder [42], but also physical symptoms such as pain [43-45]. These models can potentially be used as valuable tools for clinical diagnosis and for monitoring and altering physical responses in real time [24]. Specifically, we found a significant association between vasovagal reactions and changes in facial temperature [25] as well as facial micro-expressions [33]. Both changes in thermal fluctuations and facial action units recorded prior to blood donation showed promising results in predicting vasovagal reactions that occur during or after blood donation [24, 25, 33].”

**6. Minor and typos:
RMSEprop --> RMSProp. Proofreading is needed to improve the presentation.**

We have proofread our text and fixed the typos.

Reviewer #1

Dear Reviewer,

First of all, we would like to thank you for your valuable insights that allowed us to improve the manuscript. In what follows, we address each comment and demonstrate the changes that were made to the manuscript.

**1. How does the study align with existing theories on the interplay between psychological states, physiological responses, and the predictability of vasovagal reactions during needle-related procedures?**

Previous studies show that anticipatory anxiety and stress is associated with increased physiological changes such as increased heart rate or cortisol levels that occur at the early stage of blood donation or any other needle-related procedure and can serve as early markers of stress. It is likely that these early subtle psychological and physiological changes could be captured using psychophysiological techniques such as infrared-thermal cameras and, as shown in this experiment, using a standard RGB camera and ML techniques. We included the examples of previous findings in our introduction to elaborate more on the relationship between psychological, physiological changes and vasovagal reactions:

“Research shows that anticipatory fear, anxiety and stress and a history of previous vasovagal reactions are one of the most important risk factors for experiencing vasovagal reactions [16, 17, 10, 18, 19]. This is corroborated by studies from Hoogerwerf et al. (2018; 2017) [20, 21] who assessed psychological, hormonal and psychophysiological stress markers in donors throughout a blood donation and found that the objective stress markers already occur at a very early stage in anticipation of the needle insertion, at which time they peak. For example, the levels of systolic blood pressure and cortisol levels increased towards needle insertion and then decreased after the blood donation [20, 21]. In addition, higher systolic blood pressure and pulse rate were found in women and first-time donors, who are at the higher risk of experiencing VVR [14, 15].”

“In one of our previous studies, we mimicked a blood donation using an experimental 'virtual' rubber arm illusion, imaging the participant with an infrared thermal imaging camera. This experiment showed that changes in facial temperature could serve as early indicators for vasovagal reactions [24, 25]. Specifically, facial temperature fluctuations in the area under the nose, chin and forehead are associated with increased risk of experiencing VVR [25]. “

**2.Considering the prevalence of severe vasovagal reactions, how does the study address the challenge of training machine learning models with limited data?**

This is indeed a challenge to train machine learning models with limited data of (specifically high VVR) examples. However, we tried a few approaches to improve our model performance. First of all, as we emphasized in our introduction, we used transfer learning to overcome the limited number of samples.

“To train a model from scratch, a large amount of training data of donors with both high and low VVR scores is required, which is difficult to obtain. To overcome this limitation, transfer learning will be applied. Transfer learning is one of the machine learning techniques where a model, initially trained on one task, is used for a different task with some additional tuning.”

Second, to increase the generalization and robustness of the trained models by exposing them to a wider range of variations in the input data, we applied various transformations to the existing video data samples in the training set. We explain this in the section “Model training, validation, and evaluation”:

“Due to class imbalance and limited number of high VVR samples, we applied video data augmentation on the high VVR cases in the training data. Data augmentation is a technique used in machine learning to artificially increase the diversity of the training dataset by applying various transformations to the existing data samples. Specifically, we generated new videos by applying horizontal flips and adding some noise(Video Augmentation Library; [62]).”

**3.How does the proposed serious game intervention aim to utilize facial information obtained through video recordings, and what advantages does visible light imaging offer over infrared thermal imaging?**

We explained on how our solution would use video data obtained through front-facing camera and biofeedback mechanism to provide this personalized solution for people with needle fear and included the reference in which there is more information and examples on how the game actually works:

“As a solution for people with needle fear, while player would be immersed in the game environment, the front-facing camera is going to continuously capture the visual data of the user and estimate the likelihood of the player experiencing a vasovagal reaction. In the game, the player is going to receive visual cues such as changing background colors (dark – higher change of experiencing VVR, light – lower change) of their risk of experiencing VVR. The player has to experiment with strategies that could help him to transition from experiencing stress of fear in the context of needles to being relatively calm (more information www.ainar.io).”

Thermal and visible light imaging are fundamentally different technologies and both of them might have pros and cons. Here we discuss a few advantages of using RGB technology over thermal imaging for our application, including RGB being cheaper and more widely available:

“Although thermal cameras can be used in various light conditions including low-light or even complete-darkness and are less likely to be affected by any changes in person’s appearance, visible light imaging provide a much more detailed information about the visual appearance, including any skin changes, have higher resolution, and, more importantly, are much cheaper and more widely than thermal imaging, making them a preferred option for an intervention.”

**4.What informed the choice of pre-trained models such as Xception and ResNet152 for feature extraction, and how do they contribute to the classification and regression tasks in predicting VVR levels?**

We elaborated on our choice of the models in our introduction: “We selected ResNet152 and Xception models because they have deeper architectures than ResNet50, VGG or Inception models that can capture more complex features and patterns in data [51, 55], potentially leading to better performance in capturing subtle facial changes. In addition, ResNet152 and Xception are trained on larger datasets with more diverse images, which allows them to learn richer and more generalizable representations compared to shallower models like ResNet50 and VGG [51, 55-56].”

Both of these models are used as pre-trained models. We included this explanation on what is the advantage of using transfer learning instead of training the model from scratch: “To train a model from scratch, a large amount of training data of donors with both high and low VVR scores is required, which is difficult to obtain. To overcome this limitation, transfer learning will be applied. Transfer learning is one of the machine learning techniques where a model, initially trained on one task, is used for a different task with some additional tuning. The main advantages of transfer learning are that it requires smaller datasets and shorter computational time, and may improve the overall performance since the initial model was trained on a much larger dataset [47].”

And we specified that our aim is “to assess to what extent video classification algorithms such as Xception and ResNet152 pre-trained models with GRU and LSTM can be used to correctly classify the level of VVR a donor is experiencing.”

**5.Can you elaborate on the specific physiological changes, such as increased heart rate and changes in facial pallor, associated with vasovagal reactions?**

We include examples of specific physiological changes. For example, related to heart-rate and pulse:

“During a vasovagal reaction, the patient or the blood donor experiences a sudden drop in arterial blood pressure and as a result, it reduces blood flow to the brain and the person reports feeling lightheaded or dizzy [7]. “

<...>

“For example, the levels of systolic blood pressure and cortisol levels increased towards needle insertion and then decreased after the blood donation [20, 21]. In addition, higher systolic blood pressure and pulse rate were found in women and first-time donors, who are at the higher risk of experiencing VVR [14, 15].”

And facial temperature differences:

“Specifically, facial temperature fluctuations in the area under the nose, chin and forehead are associated with increased risk of experiencing VVR [25].”

Reviewer #2

Dear Reviewer,

We would like to thank you for valuable comments that allowed us to further improve the manuscript. In what follows, we address each comment and demonstrate the changes that were made to the manuscript.

**1. The text is written a bit carelessly. Proofread it.**

We have proofread our text and fixed some of the typos that we previously missed.

**2. The employed LSTM and GRU model parameters should be provided.**

We have included the list of all tested hyperparameters:

“For each pre-trained models with both GRU and LSTM models we have tested the following hyperparameters:

1.	Learning rate: 0.0001, 0.001, 0.01.

2.	Dropout rate: 0.1, 0.3, 0.5.

3.	Batch size: 32, 64.

4.	Epochs: 50, 100, 200.”

And reported the hyperparameters for all the best performing models across different tested folds (see S2 Table).

| Model | Number of frames | Precision | Recall | F1 | AUC-PR | MCC | Hyperparameters used |
| --- | --- | --- | --- | --- | --- | --- | --- |
| Pre-trained Xception model with GRU | N = 150 | 0.75 | 0.63 | 0.68 | 0.65 | 0.43 | 'batch_size': 64, 'dropout_rate': 0.1, 'epochs': 100, 'learning_rate': 0.001 |
|  | N = 100 | 0.74 | 0.66 | 0.70 | 0.65 | 0.46 | 'batch_size': 64, 'dropout_rate': 0.1, 'epochs': 100, 'learning_rate': 0.001 |
|  | N = 50 | 0.63 | 0.67 | 0.65 | 0.57 | 0.36 | 'batch_size': 32, 'dropout_rate': 0.1, 'epochs': 200, 'learning_rate': 0.0001 |
|  | N = 25 | 0.71 | 0.61 | 0.66 | 0.61 | 0.40 | 'batch_size': 32, 'dropout_rate': 0.5, 'epochs': 200, 'learning_rate': 0.0001 |
| Pre-trained Xception model with LSTM | N = 150 | 0.76 | 0.63 | 0.68 | 0.65 | 0.45 | 'batch_size': 64, 'dropout_rate': 0.5, 'epochs': 100, 'learning_rate': 0.0001 |
|  | N = 100 | 0.63 | 0.71 | 0.67 | 0.57 | 0.38 | 'batch_size': 64, 'dropout_rate': 0.1, 'epochs': 100, 'learning_rate': 0.0001 |
|  | N = 50 | 0.68 | 0.71 | 0.69 | 0.60 | 0.43 | 'batch_size': 64, 'dropout_rate': 0.1, 'epochs': 200, 'learning_rate': 0.0001 |
|  | N = 25 | 0.73 | 0.61 | 0.67 | 0.64 | 0.40 | 'batch_size': 32, 'dropout_rate': 0.1, 'epochs': 100, 'learning_rate': 0.0001 |
| Pre-trained ResNet152 model with GRU | N = 150 | 0.69 | 0.68 | 0.69 | 0.61 | 0.44 | 'batch_size': 64, 'dropout_rate': 0.5, 'epochs': 200, 'learning_rate': 0.001 |
|  | N = 100 | 0.75 | 0.68 | 0.71 | 0.66 | 0.48 | 'batch_size': 64, 'dropout_rate': 0.1, 'epochs': 100, 'learning_rate': 0.001 |
|  | N = 50 | 0.78 | 0.64 | 0.70 | 0.67 | 0.49 | 'batch_size': 32, 'dropout_rate': 0.5, 'epochs': 100, 'learning_rate': 0.001 |
|  | N = 25 | 0.65 | 0.79 | 0.71 | 0.61 | 0.41 | 'batch_size': 32, 'dropout_rate': 0.5, 'epochs': 200, 'learning_rate': 0.001 |
| Pre-trained ResNet152 model with LSTM | N = 150 | 0.73 | 0.81 | 0.71 | 0.60 | 0.40 | 'batch_size': 64, 'dropout_rate': 0.5, 'epochs': 100, 'learning_rate': 0.001 |
|  | N = 100 | 0.67 | 0.71 | 0.69 | 0.61 | 0.38 | 'batch_size': 64, 'dropout_rate': 0.5, 'epochs': 100, 'learning_rate': 0.001 |
|  | N = 50 | 0.75 | 0.63 | 0.69 | 0.66 | 0.44 | 'batch_size': 64, 'dropout_rate': 0.5, 'epochs': 200, 'learning_rate': 0.001 |
|  | N = 25 | 0.77 | 0.63 | 0.70 | 0.67 | 0.47 | 'batch_size': 64, 'dropout_rate': 0.1, 'epochs': 100, 'learning_rate': 0.001 |

**3. Tune the hyperparameters of used employed LSTM and GRU models for improved performance.**

Thank you for your suggestion on how we can improve the model’s performance. We have re-run our analysis using a nested 5-fold stratified cross-validation. We have employed the following hyperparameters:

“For each pre-trained models with both GRU and LSTM models we have tested the following hyperparameters:

1.	Learning rate: 0.001, 0.01.

2.	Dropout rate: 0.1, 0.3, 0.5.

3.	Batch size: 32, 64.

4.	Epochs: 50, 100, 200.”

However, the performance was similar despite the hyperparameter tuning (see S1, S3 and S3 Tables in the Supplementary Material) with SD ranging from 0.02 to 0.04 across the majority of tested folds.

**4. Although stochastic, 2D-CNN, LSTM and GRU models are employed in this paper, no statistical analysis on the obtained results are carried out.**

We performed the Friedman test to compare the performance of the models and evaluate whether there are any statistically significant differences among the best performing models.

“We completed the Friedman test to determine if there were any statistically significant differences in performance metrics (such as F1 score, precision, and recall) across four models. We selected the scores obtained using the shortest video duration on classifying the high VVR class. We found no statistically significant differences in the performance of the models (Friedman test statistic = 5.64, p = 0.6).”

**5. It is suggested to repeat the simulations and apply non-parametric statistical tests on the obtained results to draw reliable conclusions.**

Thank you for your suggestions. To ensure the robustness of our results, we have repeated the simulations and applied a 5-fold stratified validation technique on the training set, in which the dataset is divided into 5 subsets, and the model is trained and tested 5 times. In each iteration, one subset is used for testing, and the remaining 4 subsets are used for training. Stratified cross-validation was selected to ensure that each fold contains a representative sample of all classes.

Additionally, we again compared the performance of the all tested folds on this dataset using the Friedman test. This time we found that the Friedman test showed statistically significant differences between the shortest duration models (Friedman test statistic = Friedman Test Statistic: 12.12, p-value: 0.007) in F1 score across 5 tested folds. However, post-hoc tests revealed that this difference was only between pre-trained Xception model with GRU and pre-trained ResNet152 with GRU showing that ResNet152 with GRU performed statistically significant better than Xception with GRU.

Here are the obtained F1 scores across tested folds:

f1_scores_ResNetGRU = [0.6556836902800659, 0.686478454680535, 0.6760070052539404, 0.7104851330203442, 0.6737804878048781]

f1_scores_ResNetLSTM = [0.7107913669064748, 0.6601941747572816, 0.6516220028208745, 0.7038216560509554, 0.6746268656716418]

f1_scores_XceptionGRU = [0.6260869565217391, 0.6431095406360424, 0.5976562500000001, 0.6569105691056911, 0.6460032626427407]

f1_scores_XceptionLSTM = [0.6308492201039861, 0.5942857142857143, 0.6497277676950998, 0.570902394106814, 0.6656151419558359]

We believe that our reported results are in line with this finding where we concluded that pre-trained ResNet152 model yields better results than Xception.

**6. The figure quality of figure 1,2, 5 need improvement.**

We have replaced the mentioned figures.

Reviewer #3: 

Dear Reviewer,

We would like to thank you for valuable comments and questions that allowed us to further improve the manuscript. In what follows, we address each comment and demonstrate the changes that were made to the manuscript.

**1- The authors have provided an elaborate introduction. It could be made more concise and precise to offer sufficient context for the study.**

Thank you for your suggestion. We adjusted our introduction.

**2- Authors should also discuss the age range of the donors and if at all there was any difference.**

We found no statistically significant difference in age (F(2)= 1.66, p = 0.19; M = 38,98, SD = 13.45) between the groups (reported in Results/Participants section).

“No statistically significant differences in gender (F(2) = 2.76, p = .065), blood collection centers (F(2) = 1.57, p = .21), or age (F(2)= 1.66, p = 0.19; M = 38,98, SD = 13.45) were found between the groups.”

**3- Authors should elaborate on the high VVR class, and what implications do the model's performance metrics have for identifying individuals at risk of experiencing VVR symptoms?**

We have elaborated in the discussion on the selection of the model and why it was based on the performance in identifying the high VVR class:

“The best balance in classifying both groups was achieved using ResNet152 with LSTM with an MCC score of 0.61. The balance between correctly identifying the low and high VVR groups is preferred, however, given that the current model makes mistakes, the preference is to capture as many individuals at risk of experiencing high VVR symptoms even if the model sometimes incorrectly labels those who are not at risk. This is important because those who may experience vasovagal reactions, are more likely to develop needle fear and have repeat vasovagal reactions [1, 10, 14]. Thus, since ResNet152 showed a slightly higher performance in the low VVR group reaching an F1 score of 0.83, the same model showed also high performance in the high VVR group reaching an F1 score of 0.74. Therefore, we concluded that the ResNet152 with an F1 score of 0.74 on 25 frames is preferred for further investigation.”

**4- A discussion on the practical implications of the study's findings is needed. How could the identified predictive features in the nose and eye regions be practically applied? What are the potential real-world applications and benefits of using this method for predicting VVR responses?**

Indeed these are important questions for any practical solutions. We included more information on how our biofeedback solution is supposed to work using front-facing mobile phone camera and providing continuous feedback to the user:

“As a solution for people with needle fear, while player would be immersed in the game environment, the front-facing camera is going to continuously capture the visual data of the user and estimate the likelihood of the player experiencing a vasovagal reaction. In the game, the player is going to receive visual cues such as changing background colors (dark – higher change of experiencing VVR, light – lower change) of their risk of experiencing VVR. The player has to experiment with strategies that could help him to transition from experiencing stress of fear in the context of needles to being relatively calm (more information www.ainar.io).”

And we also briefly elaborated on how specific areas such as the nose and eye regions can be practically applied and what would be the benefits of using them:

“Potentially, we could focus on a smaller facial area such as eye and nose regions for extracting facial features. This potentially can provide a good model performance, and also use fewer computational resources in the real-world applications in comparison to using the facial features extracted from the entire face.”

**5-A brief mention of potential future work or directions for further research. Are there specific areas or aspects that could be explored in subsequent studies?**

We included a paragraph on some limitations of our current study and how we are planning to address them in the future studies and which areas we want to explore in subsequent studies:

“In this study, we used 2D-CNN with LSTM and GRU models and did not explore, for instance, 3D-CNN or hybrid models that could combine both spatial and temporal data streams simultaneously. Moreover, it might be worthwhile to collect psychophysiological data such as heart rate, respiration and blood pressure in order to incorporate multiple streams of information or compare their performances. Finally, as the prevalence rates of fear and VVR are higher in more general samples, it would be ideal if more data could be collected in the future among individuals who are at high risk of experiencing vasovagal reactions e.g. patients undergoing blood draws or immunizations. Some of these limitations will be remedied in the future, as we are currently repeating the virtual rubber arm experiment study. We not only use both thermal and RGB cameras, but also include psychophysiological measurements. This study will also allow us to invite people from the general population, potentially allowing us to collect a more diverse sample.”
