# Supplementary material for: Predicting vasovagal reactions to needles from video data using 2D-CNN with GRU and LSTM
Source: PLoS One. 2025 Jan 24;20(1):e0314038. doi: 10.1371/journal.pone.0314038 (PMC11760633; doi:10.1371/journal.pone.0314038)
Supplement: Attachment — Submitted filename: Response to Reviewers.docx [file pone.0314038.s003.docx]

Dear Editor and Reviewers,

We sincerely thank the new reviewer and the editor for their careful evaluation of our manuscript. We have addressed all of your suggestions for improvement, and a summary of our responses and modifications in relation to each comment is provided below (highlighted in red).

**1. In the introduction part - first paragraph, the authors mentioned “these reactions can result in physiological changes such as increased heart rate…”. Then authors mentioned parasympathetic activity such as drops in heart rate or blood pressure. It might be better to bring out only parasympathetic activity directly to avoid confusion.**

Thank you for pointing this out. Indeed this can be misleading, therefore, we adjusted the text to better explain how the vasovagal reactions occur:

“These so-called vasovagal reactions (VVR) can manifest as nausea, dizziness, heart palpitations, hyperventilation, or even fainting with a temporary loss of consciousness. They occur when the body experiences an exaggerated response to a trigger such as blood draws or injections during the medical procedure. The VVR reactions are primarily mediated by the autonomic nervous system, which controls involuntary functions like heart rate and blood pressure. Initially, the sympathetic branch of the autonomic nervous system activates in response to the stressful or fearful situation, as part of the body's "fight or flight" mechanism, leading to an increase in heart rate [2-3], sweating, nausea, and pupil dilation [4, 5, 6]. However, in some individuals, the parasympathetic nervous system, which serves as the "rest and digest" counterpart, overcompensates for this initial response, working to slow down the heart rate and lower blood pressure in an attempt to restore balance. When this parasympathetic response becomes too strong, it can cause a rapid drop in heart rate and blood pressure [3], reducing blood flow to the brain. This decrease in blood and oxygen supply can result in dizziness, nausea, and ultimately fainting (syncope) [7].”

**2. How to define VVR in case group? Are the vasovagal reactions reported by patients themselves or diagnosed by doctors or trained medical staff?**

The vasovagal reactions were self-reported by patients. We now specified this in the section called Procedure:

“Specifically, at each stage donors were recorded using a regular video camera and had to verbally self-report their VVR score.”

And under section Materials and Methods. *Vasovagal reactions*:

“participants were asked to verbally rate 8 questions regarding experienced physiological reactions”

**3. How does this quick and short-time identification method prevent VVR? Why is it important? Authors mentioned several potential ways to prevent VVR from happening, but how can identifying VVR prevent VVR? It would be good if authors could describe more on the possible application on medical perspectives.**

Thank you for your question. It seems this was not well explained in the text. We modified the “Applying deep learning methods for automated video analysis for a biofeedback-based serious game intervention” section in the Introduction, to better explain on how the game would use information of the risk of experiencing VVRs:

“In order to address the lack of interventions that address the anticipatory risk factors for VVR in practice we developed a solution for people with needle fear that is able to not only identify covert symptoms of emotional and physical reactions at a very early stage, but also to immediately give them a tool which can help them to prevent the escalation into a vasovagal event. To achieve that, we aimed to implement the best performing model in a serious game for smartphones, which through the facial video input from the front-facing camera, controls a biofeedback mechanism which will help the player get control over their impeding VVR in an early stage.

<...>

However, assessing the risk of VVR is only one part of the solution. Even better would be if the patient can use this information to prevent the VVR from happening. This can be achieved through biofeedback. <....>

By seeing or hearing these physiological signals in real time, patients can experiment with strategies to manage their stress or anxiety and bring their physiological state back into balance. <...> In these games, the stimuli adjust based on the player’s bodily responses, allowing the individual to practice managing their physiological reactions in a controlled and engaging environment.

In the case of needle fear, our serious game solution (called AINAR, Artificial Intelligence for Needle Induced Fainting) continuously assesses the likelihood of experiencing a vasovagal reaction (VVR) through the model, which gets its input from the front-facing camera, which is then reflected in the weather, which can be sunny, rainy, or snowy. The player's task is to keep the weather nice and sunny. If it starts to rain, the player can experiment with different relaxation techniques or cognitive strategies to transition from a state of fear or stress to calmness, thus learning to control their body's automatic responses. The aim is to provide the feedback as often as possible with little delay, therefore, we aim to investigate what is the shortest length of video that is required for acceptable VVR prediction. This approach allows for real-time learning and adaptation, making biofeedback a powerful tool for overcoming anxiety and stress-related conditions.”
